# That Stinging Sensation: Modularity and the Origin of the Stinging Cell

**DOI:** 10.1093/icb/icaf070

**Published:** 2025-06-12

**Authors:** Leslie S Babonis

**Affiliations:** Department of Ecology and Evolutionary Biology, Cornell University, Ithaca, NY 14853, USA

## Abstract

All cells arise through division of other cells; thus, cells with new functions (novel cell types) must evolve from ancestral cells with a different function. How, then, do novel cell types arise? Each cell is a composite of many functions that, together, confer a cell’s phenotype. A single cell might have both the capacity to sense the environment and to secrete a specialized product. Allowing those two phenotypic modules to vary independently enables the diversification of groups of cells that either secrete the same product in response to diverse environmental cues or that secrete different products in response to the same cue. In this review, I summarize the shared and unique phenotypic modules that define two types of secretory cells in cnidarians (corals, jellyfish, and their kin): mechanosensory neurons and cnidocytes (stinging cells). I then propose a series of discrete changes that could have driven the origin of a cnidocyte from an ancestral cell that looked much like a modern mechanosensory neuron. I argue that modeling cell type diversification in this way—by gain, loss, and modification of existing phenotype modules—is useful for interpreting patterns of shared gene expression across related cell types and for predicting how new cell types could arise.

## Introduction

Multicellular organisms are defined by their capacity to make many different cell types. While there are many ways to define cell “type” (Zeng 2022; Domcke and Shendure 2023), cell function is of particular value when modeling the expansion of cell type diversity, as cell function is the phenotype on which selection acts to drive the emergence of novelty (Morris 2019; Pomaville et al. 2024). The earliest multicellular organisms may have had only few cells with distinct functions, but extant organisms may have hundreds or more. Consider a single-celled organism with one cell that performs two functions separated temporally; it is not hard to imagine that a multicellular organism could evolve by duplication of the ancestral cell followed by spatial segregation of the two ancestral functions into two distinct cells (Ispolatov et al. 2012; Brunet and King 2017). Indeed, this “division of labor” model has been used to explain the gain of multicellularity in Volvicine algae (Kirk 2003) and the elaboration of the first nervous systems in animals (Arendt et al. 2015). However, this model does not explain how cells with fundamentally new functions (functions not present in the ancestral cell) arise. Taxon-specific cell functions can arise alongside the gain of new genetic material through mutation or horizontal gene transfer from another species (Boto 2014; Daugherty and Zanders 2019; Van Oss and Carvunis 2019), through reshuffling of ancestrally disparate gene networks into new combinations (McDonald and Reed 2024), or through stabilization of cell fate through chromatin imprinting (Bell et al. 2024). With access to new technologies for analyzing snapshots of gene expression in any cell from any organism at any time, it has become almost trivial to identify cell “types” by their gene expression profiles, even across wide evolutionary distances (Feregrino and Tschopp 2022). But how do we know when we observe an expected pattern of gene expression vs. something novel? Logically, it should be possible to predict which cell types will share similar gene expression profiles based on shared aspects of their phenotype. Comparing observed gene expression profiles to these predictions provides a valuable way of detecting novelty. For this reason, stepping back from gene expression and considering cell phenotype as an assemblage of discrete modules should prove a fruitful way forward in our ability to understand how extant cells came to be and to predict how new cell types could arise.

One of the innovations that accompanied the rise of animals was the origin of the specialized regulated secretory cell. These cells make and package a product into secretory vesicles that are then stored in the cytoplasm as the cell waits for a cue to release its contents (for excellent reviews of regulated secretion, see Kelly 1985; Mironov and Arvan 2008). The delay between synthesis and release of the vesicles allows the vesicles to achieve very large size in some cells (e.g., enzyme-secreting pancreatic acinar cells and mucus-secreting goblet cells), enabling the rapid release of a highly abundant product. Secretion is typically polarized, occurring across a specific part of the cellular membrane (apical or basal), and the release is not constitutive but, rather, it is regulated by input either from other cells or from the environment. The regulated secretory cells of animals use the same suite of proteins employed by unicellular eukaryotes to regulate the packaging and exocytotic release of products during constitutive secretion (Crivellato et al. 2010; Göhde et al. 2021). This suggests that regulated secretion evolved by modification of an ancestral constitutive secretion pathway and, importantly, the innovation that enabled animal cells to adopt cued release of their packaged product is what ultimately led to the evolution of the nervous system, the endocrine system, the production of a protective mucus layer, and several taxon-specific functions.

The presence of neurons in ctenophores combined with evidence of neural-like gene expression in secretory cells from sponges and placozoans (Musser et al. 2021; Najle et al. 2023) supports an ancient origin of neurosecretory cells, likely in the stem animal (Ryan and Chiodin 2015). Likewise, mucus-secreting cells are found throughout animals, suggesting the ability to secrete a protective mucus layer likely evolved alongside multicellularity in early animals (Arendt et al. 2015; McShane et al. 2021). In addition to these animal-specific cells, novel (taxon-specific) regulated secretory cells have emerged numerous times during the diversification of animals, including the array of adhesive-secreting colloblasts found among ctenophores (Leonardi et al. 2020), the diverse assemblage of cnidocytes whose secreted weaponry varies with diet specialization in cnidarians (Damian-Serrano et al. 2021), and the fast-evolving epithelial cells that secrete the exquisite variety of shells in mollusks (Batzel et al. 2022; Salamanca-Díaz et al. 2022). Many of these lineage-specific secretory cells appear to have a close developmental relationship with neurons (Babonis et al. 2018), which indicates that the origin of regulated secretion followed by repeated modification to the ancestral secretory cell phenotype has been an important mechanism for generating cell type diversity throughout the evolution of animals.

Cnidarians, the clade of animals including jellyfish, hydroids, corals, and sea anemones, have emerged as valuable model organisms for investigating the origin and diversification of novel cell types (Babonis and Martindale 2014). While they have only 10–20 morphologically discernable cell types (Burnett 1966; Frank and Bleakney 1976) assessments of cell identity using single cell RNA sequencing (scRNAseq) suggest these animals exhibit a complex repertoire of cell functions (Sebé-Pedrós et al. 2018; Siebert et al. 2019; Chari et al. 2021; Levy et al. 2021; Steger et al. 2022; Cole et al. 2024; Schnitzler et al. 2024; Salamanca-Díaz et al. 2025). These studies also demonstrate a close molecular relationship between neurons and cnidocytes, supporting early reports of a close developmental relationship between these cells in *Hydra* (David and Gierer 1974). With access to tools for manipulating gene expression in a broad diversity of cnidarian taxa, we now know that neurons, cnidocytes, and other types of secretory cells differentiate from a common population of progenitor cells in representatives of both anthozoans and medusozoans (Richards and Rentzsch 2014; Flici et al. 2017; Chrysostomou et al. 2022). Thus, the close evolutionary and developmental relationship between neurons and cnidocytes likely emerged in the stem cnidarian.

**Fig. 1:**
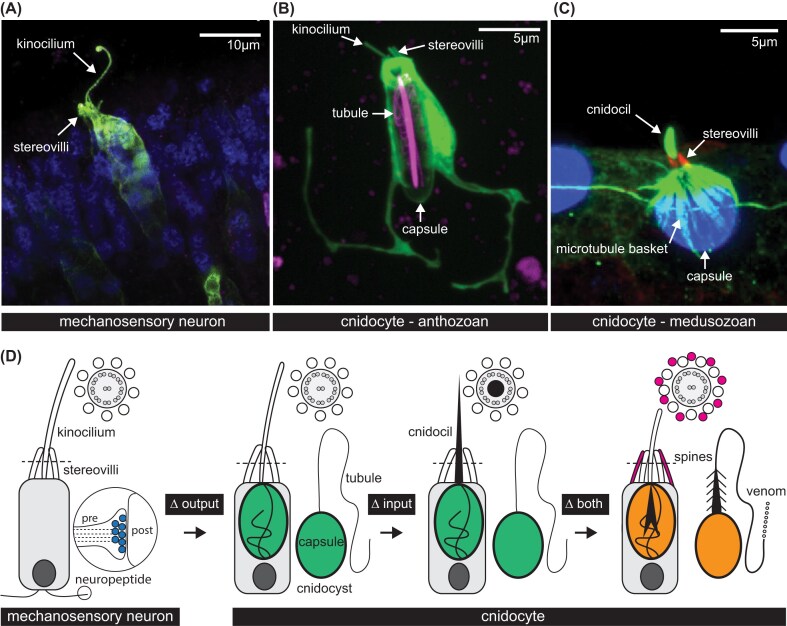
Evolution of cell type diversity by modifying phenotype modules. (A–C) Fluorescent labeling of individual cells from cnidarians highlights shared and unique phenotype modules. (D) Conceptual diagram illustrating how cell type diversity can arise in secretory cells by changing only the output of the cell from a presynaptic vesicle containing neuropeptide to a cnidocyst containing a pressurized capsule and an eversible tubule (indicated as "∆ output"), changing only the input to the cell (from a 9 + 2 type kinocilium to a cnidocil with a reinforced core (black) while keeping the cnidocyst the same (indicated as "∆ input"), or by changing both modules at the same time by gaining supporting microvilli at the apex of the cnidocyte and modifying the cnidocyst to have spines and venom (indicated as "∆ both"). In (D), dotted lines indicate a cross section through the apical sensory apparatus diagramed at the top of each cartoon showing microtubule couplets (dark gray) inside the cilium (light gray) and the surrounding stereovilli (white). Each cnidocyte is shown in unfired (left) and fired (right) state. “Pre” and “post” refer to the presynaptic cell (mechanosensory neuron) and its postsynaptic target cells. (A) modified from Tournière et al. 2020, (B) modified from Karabulut et al. (2022). Creative Commons open access license for A and B: https://creativecommons.org/licenses/by/4.0/. (C) modified from Norekian and Meech (2020) “with permission”.

**Fig. 2:**
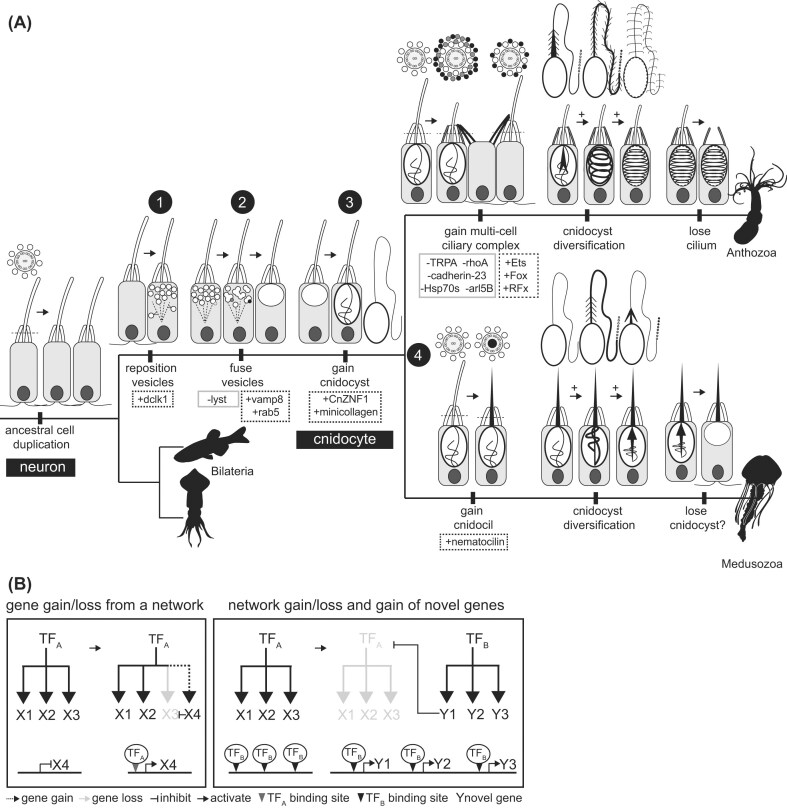
A model to describe the origin and diversification of cnidocytes by modification of an ancestral mechanosensory neuron. (A) Schematic representation of the transition from neuron to cnidocyte. Multiple types of mechanosensory neurons are present throughout cnidarians (Anthozoa + Medusozoa) and bilaterians, suggesting the ancestral cell duplicated in/before the ancestor of this clade. Step 1: Repositioning of secretory vesicles from the neurites to the cell body by gain of a novel MAP like doublecortin-like kinase (+dclk1) redirecting microtubules (dotted lines) and the vesicles they traffic (white circles) to the cell body. Step 2: Fusion of secretory vesicles to create a large secretory compartment by loss of Lyst (light gray circle), which prevents vesicle fusion, and/or gain of VAMP8 (dark gray) and Rab5 (black), which promote fusion during compound exocytosis. Step 3: Gain of a novel gene network controlled by cnidocyte-specific transcription factor CnZNF1 (formerly ZNF845) that directs the sequestration of minicollagen (and other cnidocyst-specific proteins) into a large apical secretory vesicle during formation of the cnidocyst. Step 4: Taxon-specific modifications. Anthozoa: inhibition of the genes that regulate the dynamic tuning of the apical sensory apparatus on the cnidocyte and gain of cnidocyte-specific transcription factors to drive microvilli elaboration (see text for gene references). Medusozoa: gain of a stiffened cnidocil by gain of cnidocil-specific protein nematocilin. Duplication of the ancestral cnidocyte and lineage-specific diversification of both the apical sensory apparatus and the cnidocyst would have promoted cnidocyte diversification in both clades. Loss is also an important contributor to the evolution of cell type diversity, as exemplified by the loss of the kinocilium from the spirocytes of hexacorals (see: Mariscal et al. 1976) and the origin of a new mechanosensory neuron by retention of the cnidocil and loss of the cnidocyst in a subset of hydrozoans (see: Golz and Thurm 1994). Silhouettes from phylopic.org. Cartoons as described in Fig. 1. (B) Diagram explaining the dynamic evolution of the gene networks controlling cell phenotype. Left: Networks can gain new downstream genes (dotted lines) by the origin of a new transcription factor binding site in the regulatory region of that gene (gray triangle). Gene loss (gray arrow) can be driven by mutations in the regulatory region or inhibition of gene expression by other genes (hash arrow). Right: Inhibition of transcription factors can lead to downregulation of whole networks (gray network). Novel genes can be incorporated into an existing network if they emerge from the genome in a region near an existing transcription factor binding site (black triangles).

In this article, I argue that conceptualizing a cell’s “type” as an assemblage of discrete phenotypic modules generates testable hypotheses that can both explain cases of shared gene expression across distantly related cell types and enable us to envision how new cell functions could arise through the gain, loss, and modification of individual modules. To illustrate this point, I compare the modular phenotypes of two related secretory cells in cnidarians, mechanosensory neurons and cnidocytes (Fig. 1), and propose a series of steps necessary to convert one into the other (Fig. 2). There are many thorough reviews of the form, function, and evolution of neurons and cnidocytes in cnidarians, including those evaluating the genetic control of nervous system development and evolution (Galliot et al. 2009; Watanabe et al. 2009; Rentzsch et al. 2017; Sprecher 2022; Hehmeyer et al. 2024) and those focused specifically on the morphology and physiology of these cells (Watson and Mire-Thibodeaux 1994; Tardent 1995; Kass-Simon and Scappaticci 2002; Cannon and Wagner 2003; Kass-Simon and Pierobon 2007; David et al. 2008; Anctil 2009; Anderson and Bouchard 2009; Fautin 2009; Özbek et al. 2009; Özbek 2011; Beckmann and Özbek 2012). It is not my intention to revisit those contributions here in any depth; rather, the works referenced below were selected to highlight recent contributions to our understanding of the commonalities between these secretory cell types and/or to reexamine older publications that provide new insights into the evolution of modular cell phenotype.

## Mechanosensory neurons (cnidarian hair cells)

Mechanosensory neurons are present throughout cnidarians and bilaterians and, therefore, were likely present in the last common ancestor of these clades. Indeed, evidence of vibration sensitivity in ctenophore neurons suggests this function may have arisen much earlier, in the stem animal (Horridge 1965). Similar in form and function to the hair cells of the vertebrate inner ear, the mechanosensory neurons of cnidarians have become an important model for understanding the physiology and evolution of animal sensory systems (Watson et al. 1997; Badhiwala et al. 2021; Ozment et al. 2021; Baranyk et al. 2025). Mechanosensory neurons are defined by several morphological features. At the apex of the cell, these neurons exhibit a prominent sensory apparatus that comprises a single cilium (called a “kinocilium” on mechanosensory cells) surrounded by a ring of microvilli (collectively called “stereovilli”) (Lentz and Barrnett 1965; Tardent and Schmid 1972). These stereovilli are larger in diameter than other types of microvilli and are connected to each other by protein links, i.e., tip links or intermediate links (Golz and Thurm 1991; Watson et al. 1997). Deflection of the apical sensory apparatus opens ion channels in the cell membrane causing transient intracellular current, confirming these cells are indeed mechanosensory (Arkett et al. 1988; Brinkmann et al. 1996; Watson et al. 2009). The nature of the cue these neurons secrete to regulate the behavior of other cells has not been directly revealed; however, the presence of dense-cored vesicles near where they synapse on other cells suggests the presence of neuropeptides (Koizumi et al. 1989) ; Westfall and Grimmelikhuijzen 1993 ; Westfall et al. 1998). A recent study of the sea anemone *Nematostella vectensis* showed that the density of mechanosensory neurons in the tentacles is dynamic, such that animals experiencing high rates of water flow had more mechanosensory neurons than those experiencing low/no flow (Campbell et al. 2018). In the jellyfish stage of medusozoans, pulsing rate, orientation, and swimming can all be inhibited by chemical ablation of mechanosensory cell function (McAfee et al. 2015). Together, these studies highlight the physiology of the mechanosensory neurons in cnidarians and the dynamic processes they regulate.

Cnidarians have multiple distinct types of mechanosensory neurons (Horridge 1969; Singla 1975; Hundgen and Biela 1982; Arkett et al. 1988; Golz and Thurm 1993; Westfall et al. 1998; Menard and Watson 2017; Baranyk et al. 2025), some of which have been shown to synapse on cnidocytes (Yu et al. 1985; Hobmayer et al. 1990; Mire-Thibodeaux and Watson 1994; Watson and Mire-Thibodeaux 1994; Westfall 1996; Westfall et al. 2002; Norekian and Meech 2020; Weir et al. 2020). Indeed, among anthozoans, a primary function of the mechanosensory neurons appears to be related to the regulation of cnidocyte discharge. In this clade, the large diameter stereovilli on the mechanosensory neuron are supported by numerous additional microvilli of smaller diameter, many of which emerge not from the sensory cell itself but from the adjacent support cells (Peteya 1975; Westfall et al. 1998). When N-acetylated sugars are added to the seawater surrounding the sensory cell, the small microvilli and the stereovilli they support elongate by actin polymerization, and this morphological change shifts the frequency of vibrations to which the mechanosensory cell is maximally sensitive (Watson and Hessinger 1987; Watson and Roberts 1994, 1995; Menard and Watson 2017) (Watson and Hessinger 1989; Watson and Mire-Thibodeaux 1994). These studies have been summarized in a model that describes how N-acetylated sugars shed from the exoskeleton of passing arthropods might be used to tune the tentacles of a sea anemone to maximize cnidocyte discharge (Watson and Mire-Thibodeaux 1994).

## Cnidocytes (cnidarian stinging cells)

Cnidocytes are a novel cell type found only in cnidarians. Mounted in the ectoderm, cnidocytes deliver their “sting” by rapid eversion of a hollow tubule from inside a pressurized capsule (Tardent 1995). In some cnidocytes, the eversible tubule is modified by the addition of spines to form a structure resembling a dart or harpoon. Although these harpoon-laden piercing cells are the most iconic of the “stinging cells,” the morphology of cnidocytes varies widely across species and differs with specialization for various functions, including prey capture, defense against predators, and anchoring to substrates (Mariscal 1974). The molecular regulation of cnidocyte development has been studied most extensively in *Hydra* and *N. vectensis*; in both taxa it has been shown to involve a combination of both cnidarian-specific and broadly conserved proteins (Hwang et al. 2007, 2008, 2010; Adamczyk et al. 2008, 2010; David et al. 2008; Özbek 2011; Balasubramanian et al. 2012; Beckmann et al. 2015; Rachamim et al. 2015; Babonis and Martindale 2017; Piriatinskiy et al. 2017; Shpirer et al. 2018; Sunagar et al. 2018; Babonis et al. 2022, 2023; Gahan et al. 2022; Guo et al. 2022; Garg et al. 2023). The structural proteins used in assembly of the cnidocyst, the specialized organelle comprising the capsule and its eversible tubule, are packaged into vesicles as they leave the Golgi apparatus and trafficked to the site of capsule synthesis where they fuse to create the growing cnidocyst (Garg et al. 2023; see also Beckmann and Özbek 2012 for a nice summary of the research on this topic). Some cnidocytes (but not all) contain venom and are known to express many cnidarian-specific venom proteins that are also transported to the developing cnidocyst in post-Golgi vesicles (Moran et al. 2013; Weston et al. 2013; Columbus-Shenkar et al. 2018; Ames et al. 2020; Klompen et al. 2021, 2022, 2023; Surm et al. 2024). Once cnidocyst development is complete, the organelle is mounted near the apical membrane of the cell, under a sensory apparatus where it awaits a cue to discharge.

The process of cnidocyst discharge is very similar to the exocytotic release of vesicles in other cells, particularly those engaged in communication across chemical synapses (Anderson and Bouchard 2009; Senatore et al. 2016). Stimulation of the sensory apparatus causes first a change of the pH inside the cell and then an osmotic pressure change inside the cnidocyst (Berking and Herrmann 2006; Marino et al. 2011). These processes drive eversion of the internalized tubule in a series of distinct phases, each with their own kinetics (Karabulut et al. 2022). Because cnidocytes are mounted in the ectoderm, the tissues that most often interact with the environment, these cells seem to have evolved a mechanism to inhibit discharge in response to erroneous signals, like the presence of ciliates tumbling around the outer epithelia (see Pantin 1942 and the references therein). Recent work has demonstrated that the resting membrane potential of cnidocytes is significantly more negative than that of other excitable cells (Weir et al. 2020). Cnidocytes, therefore, require greater stimulation to overcome their resting potential and drive cnidocyst discharge, though the voltage necessary for stimulation may vary across cnidocyte specialized for different functions (He et al. 2023).

Like the neurons described above, cnidocytes are intrinsically mechanosensory (Golz and Thurm 1991; Gitter et al. 1993; Brinkmann et al. 1996; Weir et al. 2020; Hofmann et al. 2021), but the way they detect and transduce mechanical/vibration cues varies across taxa. In medusozoans, both cnidocytes and mechanosensory neurons are adorned apically with a kinocilium encircled by a single ring of actin-based stereovilli. The stereovilli in both cell types are larger in diameter than other types of actin-based microvilli and are connected to each other by protein links—tip links at their apex and/or intermediate links closer to the base (Golz and Thurm 1991; Watson et al. 1997). The kinocilium of medusozoan cnidocytes—the cnidocil—appears to be specialized for transducing mechanical cues into release of the cnidocyst contents. This stiffened cilium is supported by an array of microtubules that varies in number across species (9 + N) and is reinforced at its core by a rigid filament made of a medusozoan-specific protein called nematocilin (Hwang et al. 2008). Emerging from the surface of the cnidocyte between the stereovilli and the cnidocil is a variable number of short rods or “pseudovilli” that are connected to an extensive array of microtubules that form an intracellular basket around the cnidocyst (Westfall 1970; Hausmann and Holstein 1985; Stidwill and Honegger 1989; Golz and Thurm 1993). This basket of microtubules, its crown of pseudovilli/rods, and the stiffened cnidocil are all unique to medusozoans, suggesting these features emerged in the stem medusozoan after this clade split from its common origin with anthozoans.

Although the ancestral cnidocyte was likely mechanosensory, the sensory apparatus of anthozoan cnidocytes has specialized in a very different way than that of medusozoans. Unlike the reinforced cnidocil of medusozoans, anthozoan cnidocytes have a flexible kinocilium with the typical 9 + 2 arrangement of microtubules, much like cnidarian mechanosensory neurons. In some anthozoan cnidocytes (e.g., mastigophores), the kinocilium is encircled by a single ring of large diameter stereovilli, much like the cnidocytes of medusozoans (Westfall et al. 1998). Other types of cnidocytes found in anthozoans vary more dramatically in the morphology of their apical sensory apparatus. In basitrichous isorhizas, the stereovilli are uniformly small in diameter and far more numerous than in mastigophores (Westfall et al. 1998), and in spirocytes, which are found only among anthozoans, the kinocilium appears to have been lost completely (Mariscal et al. 1976). In perhaps all anthozoan cnidocytes studied so far, the apical sensory apparatus is further supported by a large and variable number of microvilli emerging from adjacent support cells, creating a multi-cell ciliary complex. These support cells contribute a variable number of microvilli to both the cnidocyte and an adjacent mechanosensory neuron, and it is the elongation of the latter in response to N-acetylated sugars has been shown to play an important role in potentiating cnidocyte discharge (Mire-Thibodeaux and Watson 1994). Thus, while the cnidocytes of medusozoans are themselves mechanosensory and chemosensory (Thurm et al. 2004), these sensory modalities seem to have been outsourced to the adjacent support cells in anthozoans. Modulation of cnidocyte discharge by nearby sensory cells seems to be an ancient feature of cnidarians, as the stem cnidocyte was likely responsive to photosensory cues (Picciani et al. 2021). In some hydrozoans, this association between cnidocytes and the neurons that modulate them is taken to the extreme as cnidocytes are mounted together with the photosensory neurons in a specialized cell cluster called a battery complex (Holstein 2012; Plachetzki et al. 2012; Birch and Plachetzki 2023). At the other extreme, in the neuron-free parasitic cnidarians called myxozoans, the polar capsules (myxozoan-specific cnidocytes) appear to discharge exclusively as independent effectors without neural input (Cannon and Wagner 2003).

Recent evidence using transgenic animals has demonstrated the presence of basal projections resembling neurites extending out from the basal pole of the cnidocytes in *N. vectensis* (Sebé-Pedrós et al. 2018; Ozment et al. 2021; Karabulut et al. 2022). At present it is unclear how widespread this aspect of cnidocyte morphology may be, but this observation supports previous anatomical reports of such basal projections (Anderson and Mckay 1987) and reports of cnidocytes exhibiting presynaptic control of other cnidocytes (Brinkmann et al. 1996; Holtmann and Thurm 2001; Thurm et al. 2004). This provides evidence that cnidocytes might regulate each other like neurons do (Oliver et al. 2008; Weir et al. 2020) and could explain previous observations of cnidocyte discharge becoming inhibited when animals have reached satiety (Burnett et al. 1960; Smith et al. 1974; Thorington et al. 2010).

### From neuron to cnidocyte: a model

That mechanosensory neurons and cnidocytes share aspects of their morphology and function is not a new idea (Tardent and Schmid 1972; Westfall et al. 1998). Likewise, the idea that these two cell types are evolutionarily closely related has been suggested many times (Brinkmann et al. 1996; Galliot et al. 2009; Bosch et al. 2017; Rentzsch et al. 2019). What is lacking is a model of the process by which the various aspects of cnidocyte phenotype might have evolved from an ancestral cell without such phenotypes. For an organism to gain a new cell type without loss of an ancestral cell type, the ancestral cell lineage must first be duplicated (Serb and Oakley 2005; Babonis et al. 2022). Much like the concept of neofunctionalization as applied to gene duplication, duplication of a cell frees one cell to acquire new functions while the ancestral functions remain intact in the other. Allowing different aspects of cell phenotype to vary in distinct daughters after many rounds of duplication is an effective way to generate diversity within a lineage of closely related cells (Fig. 1). Below, I propose a series of changes that could have occurred after duplication of an ancestral mechanosensory neuron to give rise to the ancestral cnidocyte. Specifically, I propose four primary changes, including (1) gain of a mechanism for repositioning post-Golgi secretory vesicles to the cell body, (2) gain of a mechanism promoting intracellular fusion of secretory vesicles, (3) the gain of a novel secretory product (the cnidocyst), and (4) taxon-specific modifications to the apical sensory apparatus and cnidocyst that resulted in the impressive diversity of cnidocytes found among modern cnidarians (Fig. 2).

Step 1 *Gain of a mechanism for repositioning post-Golgi secretory vesicles to the cell body.* Two important aspects of neuronal phenotype are the ability of these cells to make neurites, membrane projections that permit direct cell-cell contact across long distances, and their ability to transmit information to other cells through the release of synaptic vesicles along these neurites. Neurites are supported by an extensive network of microtubules that are dynamic in their size and arrangement allowing for maximal flexibility while maintaining function during regeneration and in response to variable growth conditions (Havrilak et al. 2021; Lovas and Yuste 2021; Keramidioti et al. 2024). The microtubules also provide a conduit by which vesicles of different types can be differentially targeted to different parts of the neuron (for a nice review, see Aiken and Holzbaur 2021). Like other membrane-bound organelles, synaptic vesicles develop from the Golgi apparatus in the cell body. To get from the cell body to the synapse, these vesicles are trafficked along the microtubules with the help of motor proteins (e.g., kinesin and dynein) and other non-motor microtubule associated proteins (MAPs). Vesicles of different types are associated with unique assemblages of motor proteins and MAPs, which provide the cell with flexibility in controlling the organization of the intracellular environment (Maday et al. 2014). This suggests that the origin of novel MAPs could facilitate trafficking of new vesicle types and drive the diversification of intracellular vesicles.

Consistent with the idea that cell type specific vesicles are trafficked along microtubules by vesicle-specific MAPs, a recent study identified a cnidocyte-specific MAP required for proper cnidocyst development (Kraus et al. 2024). This gene, a doublecortin-like kinase (*dclk1*), is expressed in both neurons and cnidocytes but knockdown affected only the morphology of the cnidocytes. Several studies have shown that microtubules are important for scaffolding the vesicles while they fuse during capsule and tubule formation in developing cnidocytes (see above). Given that cnidocytes have also been shown to have presynaptic regulatory function (of other cnidocytes), it is likely that the association of doublecortin-like kinase with microtubules arose after the ancestral cnidocyte already had the ability to traffic vesicles to the membrane for synaptic secretion. Previous studies of synaptic morphology have demonstrated that cnidarian neurons make multiple different types of vesicles with distinct contents (Westfall 1973), suggesting the ancestral cnidarian neuron was multifunctional (Grimmelikhuijzen and Westfall 1995). Together, these results demonstrate how duplication of an ancestral neuron and subsequent partitioning of motor proteins and MAPs with different functions into each daughter cell could have been instrumental for the early diversification of mechanosensory cells in the stem cnidarian.

Step 2 *Gain of a mechanism promoting intracellular fusion of secretory vesicles.*The discharge of the pressurized cnidocyst capsule has been described as an exocytotic event stimulated by the influx of calcium, much like the exocytotic release of synaptic contents in neurons (Senatore et al. 2016). Exocytosis begins with influx of calcium across the cnidocyte membrane through voltage-gated calcium channels, causing docking of the vesicle to the plasma membrane. In other exocytotic cells, the influx of calcium stimulates fusion of the vesicle to the membrane by driving physical interactions between proteins localized to the cell and vesicle membranes. This process involves several proteins conserved in exocytotic cells across animals, including SNAREs (soluble N-ethylmaleimide-sensitive factor attachment protein receptors) like SNAP-23 and SNAP-25, synaptotagmin, Munc-18, syntaxins, and several VAMPs (vesicle associate membrane proteins) like synaptobrevin (VAMP2) and endobrevin (VAMP8). A search of the mRNAs expressed in different cell types in *N. vectensis* (Steger et al. 2022; Cole et al. 2024) suggests these exocytotic control genes are indeed expressed in cnidocytes.

One feature that makes cnidocytes unique among exocytotic cells is that rather than having numerous small vesicles that fuse with the plasma membrane for targeted release of neurotransmitters or neuropeptides, there is a single large vesicle that secretes an eversible tubule and its soluble contents (e.g., venom). An important step in the evolution of the cnidocyst from a cell that may have produced many small vesicles like modern neurons would have involved the gain of proteins that facilitated intracellular vesicle fusion, like cells that undergo compound exocytosis (e.g., pancreatic acinar cells, mast cells, and mucous cells; see Pickett and Edwardson 2006 for a review). Several studies of post-Golgi vesicle development shed light on the evolution of compound exocytosis. One shows that knockout of VAMP8 dramatically reduced intracellular fusion of vesicles in mouse pancreatic acinar cells (Thorn and Gaisano 2012); another showed that the GTPase Rab5 is also essential for vesicle fusion during compound exocytosis (Klein et al. 2017). By contrast, mast cells isolated from mice exhibiting a loss of function mutation for the Lyst gene, which regulates lysosomal trafficking, exhibited abnormally large vesicles, suggesting Lyst may inhibit vesicle fusion (Hammel et al. 2010). One might therefore hypothesize the gain of novel or constitutively active forms of VAMP8 or Rab5 and/or loss of Lyst expression from a duplicated neuron could have promoted the evolution of a single large intracellular vesicle. Considering this, one might expect examples of shared gene expression between cnidocytes and mucus-secreting cells or other types of gland cells in cnidarians to reflect shared expression of the machinery regulating vesicle fusion for compound exocytosis.

Step 3 *Gain of a novel secretory product (the cnidocyst).* The origin of the cnidocyst was an important step in the evolution of a cnidocyte from an ancestral mechanosensory cell. The contents of the cnidocyst—the capsule, its tubule, and their associated proteins—are made, largely, from proteins that are found only in cnidarians. In *N. vectensis*, a single transcription factor called CnZNF1 (formerly ZNF845) activates the expression of all known downstream cnidocyst-specific genes; these include structural proteins, like minicollagen, which are necessary for construction of the capsule and tubule (David et al. 2008; Zenkert et al. 2011), and a suite of other regulatory genes, including PaxA, that differentially control the development of cnidocyte subtypes (Babonis and Martindale 2017). CnZNF1 is itself cnidarian-specific; the DNA binding domain of this protein comprises a series of 6 tandemly-arrayed C_2_H_2_ zinc fingers, which were assembled de novo by exon shuffling in the stem cnidarian (Babonis et al. 2022). While the origin of this novel regulatory gene may have driven the earliest steps in the coordinated development of early cnidocyst proteins, sometime during the evolution of cnidocytes, CnZNF1 gained another important function. Knockdown of this gene in *N. vectensis* resulted in the gain of additional RFamide-expressing neurons, suggesting CnZnf1 has dual roles in driving the development of cnidocytes and also suppressing the identity of the ancestral neuron.

The C_2_H_2_ family of transcription factors arose before the origin of animals and is among the largest family of transcription factors encoded in nearly every animal genome examined so far. Thus, the genome of the stem cnidarian likely encoded many C_2_H_2_ ZNF transcription factor binding sites before the origin of CnZNF1. If the cnidocyst-specific genes unique to cnidarians (e.g., minicollagen, nematocilin) emerged from regions of the genome near existing C_2_H_2_ ZNF transcription factor binding sites, they could easily have become integrated into the CnZNF1-specific gene network after the origin of this gene. While it may not be possible to determine the order in which these cnidocyte-specific genes arose, it seems likely that sequestration of novel proteins into a secretory vesicle could protect these products from targeted degradation by the cell until they have time to acquire adaptive value. If true, one might expect regulated secretory cells to express a higher proportion of novel protein-coding effector genes than other cell types. This hypothesis should be relatively easy to test and will go a long way toward the development of a theory about how novel proteins drive the evolution of a novel organelle.

Step 4 *Taxon-specific modifications to the apical sensory apparatus and cnidocyst.* In anthozoans, specialization of the apical sensory apparatus on cnidocytes has led to the evolution of the multi-cell ciliary complex, in which the chemosensory module is outsourced to the adjacent support cell and the mechanosensory module is outsourced to a nearby neuron. Previous studies using protein immunolocalization and pharmacological inhibitors of protein function have identified several key proteins localized uniquely to the stereovilli of the mechanosensory neurons controlling cnidocyte discharge in sea anemones. These include *TRPA1*, which is the ion channel thought to transduce the vibration dependent signal into increased cnidocyte discharge (Mahoney et al. 2011), *Cadherin-23*, an integral component of the tip links that connect stereovilli in the apical sensory apparatus on these neurons (Tang and Watson 2014), and the G protein *RhoA*, which regulates the length of the stereovilli in response to chemical cues (Allaire and Watson 2013). Several proteins associated with repair of the sensory apparatus in vertebrate hair cells have also been localized uniquely to the stereovilli of the mechanosensory neuron. Inhibition of Hsp70s and several subunits of the 20S proteasome, identified from a proteomic screen of mechanosensory cells, delayed sensory apparatus recovery following injury (Tang and Watson 2015), and ARL-5B (an ADP ribosylation factor) was shown to restore the organization of the stereovilli following damage (Watson et al. 2007). Consistent with observations that mechanosensory neurons dynamically tune to the vibrations of passing prey to maximize cnidocyte discharge (see above), the stereocilia on these mechanosensory cells are also sensitive to cytochalasin (an inhibitor of actin polymerization), whereas the stereovilli of other mechanosensory cells and cnidocytes are not (Menard and Watson 2017). Importantly, all these features are common to mechanosensory neurons in other taxa, thus; if they are missing from cnidocytes in anthozoans, this likely represents a secondary loss associated with the evolution of the multi-cell ciliary complex found in this lineage. Medusozoans do not exhibit these multi-cell ciliary complexes and have likely retained these proteins in the stereocilia of the cnidocyte itself.

The evolution of the multi-cell ciliary complex in anthozoans was also likely associated with a gain of the ability of these cells to make more extensive membrane extensions in the form of supporting microvilli; however, the genes that control this process are not currently known. Transcription factors in the E26 (Ets), Forkhead box (Fox), and Regulatory Factor X (RFX) families, among others, are important for the development of apical membrane specializations (microvilli and cilia) throughout animals (Jedlicka et al. 2009; Thomas et al. 2010; Coyle et al. 2023; Ansel et al. 2024). Orthologs of these genes expressed uniquely or predominantly in cnidocytes would be good targets for investigating how these cells acquired their expansive apical sensory apparatus. If cnidocytes evolved from an ancestral mechanosensory cell, both cell types can be expected to share the expression of a suite of genes that regulate shared aspects of their phenotype. The bHLH gene *Atonal* is known to regulate the development of the mechanosensory cells in a wide variety of taxa (Jarman and Groves 2013). In *N. vectensis*, knockdown of this gene results in loss of mature sensory neurons and fully differentiated cnidocytes (Richards and Rentzsch 2015). Similarly, *POUIV/Brain3* is expressed in both mechanosensory neurons and cnidocytes, and knockdown of *POUIV* disrupts terminal differentiation of both cell types, including the formation of the apical sensory apparatus (Tournière et al. 2020; Ozment et al. 2021). This suggests *Atonal* together with *POUIV* may coordinate the expression of genes necessary for shared features in these two cell types, like the kinocilium and stereovilli. Thus, retention of the *Atonal/POUIV*-regulated apical structures in both cell types, gain of additional microvilli through cell-specific expression of transcription factors controlling apical membrane expansion in cnidocytes, and loss of the proteins necessary for transducing mechanosensory information in the cnidocyte only, could be a path toward the evolution of the multi-cell ciliary complex in anthozoans.

In medusozoans, apical specialization of the cnidocyte followed a different path. Unique to medusozoans, the cnidocil likely arose by gain of nematocilin, which forms the stiffened filament at the center of the sensory cilium (see above). An unusual type of sensory neuron in the hydrozoan *Stauridiosarsia producta* also appears to exhibit the stiffened cnidocil usually restricted to cnidocytes (Tardent and Schmid 1972; Golz and Thurm 1994). This result could reflect the evolution of a new neuron by duplication of an ancestral cnidocyte followed by loss of the cnidocyst. Alternatively, it is possible that nematocilin was independently co-opted into the regulatory program of a cell that was already a mechanosensory neuron. The presence of a large apical vacuole reminiscent of an empty cnidocyst in these cells suggests perhaps the former hypothesis is favorable. Studies of shared gene expression through single-cell sequencing paired with analysis of the developmental origin of this cell type would be informative for evaluating these two hypotheses and reconstructing how this novel neuron type arose.

## Conclusions

Understanding the mechanisms that generate cell type diversity is an ongoing challenge in evolutionary biology but one that is imminently important for developing a deeper understanding of the factors that promote and limit the evolution of biodiversity. Here, I have put forth a model to explain the evolution of a novel cell type by duplication and divergence of an ancestral cell. Importantly, the mechanosensory neuron used as the starting point in this model may not perfectly phenocopy the cells from which cnidocytes arose. Likewise, our understanding of what the first cnidocyte might have looked like is limited, despite recent efforts to reconstruct the ancestral cell from analysis of extant cnidocyte diversity (Sierra and Gold 2024). Still, organizing the evolution of a novel cell phenotype into a series of discrete changes, as I have done here, is a useful way of generating hypotheses about the mechanisms promoting cell type diversification. Applying this approach to other closely related cell types will be fruitful for developing a comprehensive understanding of the relationship between the types of changes that can occur during cell differentiation and the processes that might have promoted the emergence of cells with unique phenotypes.

Where do we go from here? Those of us with access to the organisms and the tools to manipulate cell phenotype should ask: How much must I sweep away from the identity of two related cell types until they are no longer distinguishable? Is this state even possible to achieve? For some cells, the answer is probably yes. For others, it seems likely that the evolutionary distance between them may be too great—allowing for too many ancillary mutations and module modifications—to ever really wipe away diversity and homogenize cell identity. Despite that, putting effort into this approach is perhaps the most valuable way to push forward our understanding of the actual changes that drive cell type diversification and begin to develop a theory to predict which cell phenotypes could never evolve.

## Notes

“From the symposium “Cnidarian sensory systems as comparative models for the evolution of complexity" presented at the annual meeting of the Society for Integrative and Comparative Biology, January 3-7th , 2025.''

## References

[bib1] Adamczyk P, Meier S, Gross T, Hobmayer B, Grzesiek S, Bächinger HP, Holstein TW, Özbek S. 2008. Minicollagen-15, a novel minicollagen isolated from Hydra, forms tubule structures in nematocysts. J Mol Biol. 376:1008–20. 18206162 10.1016/j.jmb.2007.10.090

[bib2] Adamczyk P, Zenkert C, Balasubramanian PG, Yamada S, Murakoshi S, Sugahara K, Hwang JS, Gojobori T, Holstein TW, Özbek S. 2010. A non-sulfated chondroitin stabilizes membrane tubulation in cnidarian organelles. J Biol Chem. 285:25613–23. 20538610 10.1074/jbc.M110.107904 PMC2919125

[bib3] Aiken J, Holzbaur ELF. 2021. Cytoskeletal regulation guides neuronal trafficking to effectively supply the synapse. Curr Biol. 31:R633–50. 34033795 10.1016/j.cub.2021.02.024 PMC8360495

[bib4] Allaire KM, Watson GM. 2013. Rho participates in chemoreceptor-induced changes in morphology to hair bundle mechanoreceptors of the sea anemone, *Nematostella vectensis*. Comp Biochem Physiol A: Mol Integr Physiol. 165:139–48. 23474255 10.1016/j.cbpa.2013.03.003

[bib5] Ames CL, Klompen AML, Badhiwala K, Muffett K, Reft AJ, Kumar M, Janssen JD, Schultzhaus JN, Field LD, Muroski ME et al. 2020. Cassiosomes are stinging-cell structures in the mucus of the upside-down jellyfish *Cassiopea xamachana*. Commun Biol. 3:67. 32054971 10.1038/s42003-020-0777-8 PMC7018847

[bib6] Anctil M . 2009. Chemical transmission in the sea anemone *Nematostella vectensis*: a genomic perspective. Comp Biochem Physiol D. 4:268–89. 10.1016/j.cbd.2009.07.001 20403752

[bib7] Anderson PAV, Bouchard C. 2009. The regulation of cnidocyte discharge. Toxicon. 54:1046–53. 19268492 10.1016/j.toxicon.2009.02.023

[bib8] Anderson PAV, Mckay MC. 1987. The electrophysiology of cnidocytes. J Exp Biol. 133:215–30.

[bib9] Ansel M, Ramachandran K, Dey G, Brunet T. 2024. Origin and evolution of microvilli. Biol Cell. 116:e2400054. 39233537 10.1111/boc.202400054

[bib10] Arendt D, Benito-Gutierrez E, Brunet T, Marlow H. 2015. Gastric pouches and the mucociliary sole: setting the stage for nervous system evolution. Phil Trans R Soc B. 370:20150286. 26554050 10.1098/rstb.2015.0286 PMC4650134

[bib11] Arkett SA, Mackie GO, Meech RW. 1988. Hair cell mechanoreception in the jellyfish. 135:329–342.

[bib12] Babonis LS, DeBiasse MB, Francis WR, Christianson LM, Moss AG, Haddock SHD, Martindale MQ, Ryan JF. 2018. Integrating embryonic development and evolutionary history to characterize tentacle-specific cell types in a ctenophore. Mol Biol Evol. 35: 2940–2956. 30169705 10.1093/molbev/msy171 PMC6278862

[bib13] Babonis LS, Enjolras C, Reft AJ, Foster BM, Hugosson F, Ryan JF, Daly M, Martindale MQ. 2023. Single-cell atavism reveals an ancient mechanism of cell type diversification in a sea anemone. Nat Commun. 14:885. 36797294 10.1038/s41467-023-36615-9 PMC9935875

[bib14] Babonis LS, Enjolras C, Ryan JF, Martindale MQ. 2022. A novel regulatory gene promotes novel cell fate by suppressing ancestral fate in the sea anemone *Nematostella vectensis*. Proc Natl Acad Sci USA. 119:e2113701119. 35500123 10.1073/pnas.2113701119 PMC9172639

[bib15] Babonis LS, Martindale MQ. 2014. Old cell, new trick? Cnidocytes as a model for the evolution of novelty. Integr Comp Biol. 54:714–22. 24771087 10.1093/icb/icu027 PMC4817572

[bib16] Babonis LS, Martindale MQ. 2017. PaxA, but not PaxC, is required for cnidocyte development in the sea anemone *Nematostella vectensis*. EvoDevo. 8:14. 28878874 10.1186/s13227-017-0077-7 PMC5584322

[bib17] Badhiwala KN, Primack AS, Juliano CE, Robinson JT. 2021. Multiple neuronal networks coordinate Hydra mechanosensory behavior. eLife. 10:e64108. 34328079 10.7554/eLife.64108 PMC8324302

[bib18] Balasubramanian PG, Beckmann A, Warnken U, Schnölzer M, Schüler A, Bornberg-Bauer E, Holstein TW, Özbek S. 2012. Proteome of Hydra nematocyst. J Biol Chem. 287:9672–81. 22291027 10.1074/jbc.M111.328203 PMC3323026

[bib19] Baranyk J, Malir K, Silva MAP, Rieck S, Scheve G, Nakanishi N. 2025. Structural, molecular and developmental evidence for cell-type diversity in cnidarian mechanosensory neurons. Nat Commun. 16:1514. 39929800 10.1038/s41467-025-56115-2 PMC11811123

[bib20] Batzel GO, Moreno BK, Lopez LS, Nguyen CK, Livingston BT, Joester D, Lyons DC. 2022. Proteomic and transcriptomic analyses in the slipper snail *Crepidula fornicata* uncover shell matrix genes expressed during adult and larval biomineralization. Integr Org Biol. 4:obac023. 35968217 10.1093/iob/obac023 PMC9365450

[bib21] Beckmann A, Özbek S. 2012. The nematocyst: a molecular map of the cnidarian stinging organelle. Int J Dev Biol. 56:577–82. 22689365 10.1387/ijdb.113472ab

[bib22] Beckmann A, Xiao S, Müller JP, Mercadante D, Nüchter T, Kröger N, Langhojer F, Petrich W, Holstein TW, Benoit M et al. 2015. A fast recoiling silk-like elastomer facilitates nanosecond nematocyst discharge. BMC Biol. 13:3. 25592740 10.1186/s12915-014-0113-1 PMC4321713

[bib23] Bell CC, Faulkner GJ, Gilan O. 2024. Chromatin-based memory as a self-stabilizing influence on cell identity. Genome Biol. 25:320. 39736786 10.1186/s13059-024-03461-x PMC11687074

[bib24] Berking S, Herrmann K. 2006. Formation and discharge of nematocysts is controlled by a proton gradient across the cyst membrane. Helgol Mar Res. 60:180–8.

[bib25] Birch S, Plachetzki D. 2023. Multisensory integration by polymodal sensory neurons dictates larval settlement in a brainless cnidarian larva. Mol Ecol. 32:3892–907. 37161896 10.1111/mec.16968

[bib26] Bosch TCG, Klimovich A, Domazet-Lošo T, Gründer S, Holstein TW, Jékely G, Miller DJ, Murillo-Rincon AP, Rentzsch F, Richards GS et al. 2017. Back to the basics: cnidarians Start to Fire. Trends Neurosci. 40:92–105. 28041633 10.1016/j.tins.2016.11.005 PMC5285349

[bib27] Boto L . 2014. Horizontal gene transfer in the acquisition of novel traits by metazoans. Proc Biol Sci. 281:20132450. 24403327 10.1098/rspb.2013.2450 PMC3896011

[bib28] Brinkmann M, Oliver D, Thurm U. 1996. Mechanoelectric transduction in nematocytes of a hydropolyp (Corynidae). J Comp Physiol A. 178: 125–138.

[bib29] Brunet T, King N. 2017. The origin of animal multicellularity and cell differentiation. Dev Cell. 43:124–40. 29065305 10.1016/j.devcel.2017.09.016 PMC6089241

[bib31] Burnett AL, Lentz T, Warren M. 1960. The question of control of nematocyst discharge reaction by fully fed Hydra. Ann Soc Roy Zool Belg. 90:247–67.

[bib30] Burnett AL . 1966. A model of growth and cell differentiation in Hydra. Am Nat. 100:165–89.

[bib32] Campbell A, Dykes A, Mire P. 2018. Periodic, moderate water flow reversibly increases hair bundle density and size in *Nematostella vectensis*. J Exp Biol. 221:jeb181081. 30397171 10.1242/jeb.181081

[bib33] Cannon Q, Wagner E. 2003. Comparison of discharge mechanisms of cnidarian cnidae and myxozoan polar capsules. Rev Fish Sci. 11:185–219.

[bib34] Chari T, Weissbourd B, Gehring J, Ferraioli A, Leclère L, Herl M, Gao F, Chevalier S, Copley RR, Houliston E et al. 2021. Whole-animal multiplexed single-cell RNA-seq reveals transcriptional shifts across *Clytia* medusa cell types. Sci Adv. 7:eabh1683. 34826233 10.1126/sciadv.abh1683 PMC8626072

[bib35] Chrysostomou E, Flici H, Gornik SG, Salinas-Saavedra M, Gahan JM, McMahon ET, Thompson K, Hanley S, Kilcoyne M, Schnitzler CE et al. 2022. A cellular and molecular analysis of SoxB-driven neurogenesis in a cnidarian. eLife. 11:e78793. 35608899 10.7554/eLife.78793 PMC9173746

[bib36] Cole AG, Steger J, Hagauer J, Denner A, Ferrer Murguia P, Knabl P, Narayanaswamy S, Wick B, Montenegro JD, Technau U. 2024. Updated single cell reference atlas for the starlet anemone *Nematostella vectensis*. Front Zool. 21:8. 38500146 10.1186/s12983-024-00529-z PMC10946136

[bib37] Columbus-Shenkar YY, Sachkova MY, Macrander J, Fridrich A, Modepalli V, Reitzel AM, Sunagar K, Moran Y. 2018. Dynamics of venom composition across a complex life cycle. eLife. 7:e35014. 29424690 10.7554/eLife.35014 PMC5832418

[bib38] Coyle MC, Tajima AM, Leon F, Choksi SP, Yang A, Espinoza S, Hughes TR, Reiter JF, Booth DS, King N. 2023. An RFX transcription factor regulates ciliogenesis in the closest living relatives of animals. Curr Biol. 33:3747–3758.e9. 37552984 10.1016/j.cub.2023.07.022 PMC10530576

[bib39] Crivellato E, Nico B, Gallo VP, Ribatti D. 2010. Cell secretion mediated by granule-associated vesicle transport: a glimpse at evolution. Anat Rec. 293:1115–24. 10.1002/ar.21146 20340095

[bib40] Damian-Serrano A, Haddock SHD, Dunn CW. 2021. The evolution of siphonophore tentilla for specialized prey capture in the open ocean. Proc Natl Acad Sci USA. 118:e2005063118. 33593896 10.1073/pnas.2005063118 PMC7923536

[bib41] Daugherty MD, Zanders SE. 2019. Gene conversion generates evolutionary novelty that fuels genetic conflicts. Curr Opin Genet Dev. 58–59:49–54. 10.1016/j.gde.2019.07.011 PMC6889005 31466040

[bib42] David CN, Gierer A. 1974. Cell cycle kinetics and development of Hydra attenuata: III. nerve and nematocyte differentiation. J Cell Sci. 16:359–375. 4448826 10.1242/jcs.16.2.359

[bib43] David CN, Özbek S, Adamczyk P, Meier S, Pauly B, Chapman J, Hwang JS, Gojobori T, Holstein TW. 2008. Evolution of complex structures: minicollagens shape the cnidarian nematocyst. Trends Genet. 24:431–8. 18676050 10.1016/j.tig.2008.07.001

[bib44] Domcke S, Shendure J. 2023. A reference cell tree will serve science better than a reference cell atlas. Cell. 186:1103–14. 36931241 10.1016/j.cell.2023.02.016

[bib45] Fautin DG . 2009. Structural diversity, systematics, and evolution of cnidae. Toxicon. 54:1054–64. 19268491 10.1016/j.toxicon.2009.02.024

[bib46] Feregrino C, Tschopp P. 2022. Assessing evolutionary and developmental transcriptome dynamics in homologous cell types. Dev Dyn. 251:1472–89. 34114716 10.1002/dvdy.384 PMC9545966

[bib47] Flici H, Schnitzler CE, Millane RC, Govinden G, Houlihan A, Boomkamp SD, Shen S, Baxevanis AD, Frank U. 2017. An evolutionarily conserved SoxB-Hdac2 crosstalk regulates neurogenesis in a cnidarian. Cell Rep. 18:1395–409. 28178518 10.1016/j.celrep.2017.01.019 PMC5312794

[bib48] Frank P, Bleakney JS. 1976. Histology and sexual reproduction of the anemone *Nematostella vectensis* Stephenson 1935. J Nat Hist. 10:441–9.

[bib49] Gahan JM, Kouzel IU, Jansen KO, Burkhardt P, Rentzsch F. 2022. Histone demethylase Lsd1 is required for the differentiation of neural cells in *Nematostella vectensis*. Nat Commun. 13:465. 35075108 10.1038/s41467-022-28107-z PMC8786827

[bib50] Galliot B, Quiquand M, Ghila L, de Rosa R, Miljkovic-Licina M, Chera S. 2009. Origins of neurogenesis, a cnidarian view. Dev Biol. 332:2–24. 19465018 10.1016/j.ydbio.2009.05.563

[bib51] Garg N, Štibler UK, Eismann B, Mercker M, Bergheim BG, Linn A, Tuchscherer P, Engel U, Redl S, Marciniak-Czochra A et al. 2023. Non-muscle myosin II drives critical steps of nematocyst morphogenesis. iScience. 26:106291. 36936784 10.1016/j.isci.2023.106291 PMC10014300

[bib52] Gitter AH, Oliver D, Thurm U. 1993. Streptomycin inhibits nematocyte discharge inHydra vulgaris by blockage of mechanosensitivity. Naturwissenschaften. 80:273–6.

[bib53] Göhde R, Naumann B, Laundon D, Imig C, McDonald K, Cooper BH, Varoqueaux F, Fasshauer D, Burkhardt P. 2021. Choanoflagellates and the ancestry of neurosecretory vesicles. Phil Trans R Soc B. 376:20190759. 33550951 10.1098/rstb.2019.0759 PMC7934909

[bib54] Golz R, Thurm U. 1991. Cytoskeleton-membrane interactions in the cnidocil complex of hydrozoan nematocytes. Cell Tissue Res. 263:573–83.

[bib55] Golz R, Thurm U. 1993. Ultrastructural evidence for the occurrence of three types of mechanosensitive cells in the tentacles of the cubozoan polypCarybdea marsupialis. Protoplasma. 173:13–22.

[bib56] Golz R, Thurm U. 1994. The ciliated sensory cell of *Stauridiosarsia producta* (Cnidaria, Hydrozoa)—a nematocyst-free nematocyte?. Zoomorphology. 114:185–94.

[bib57] Grimmelikhuijzen CJP, Westfall JA. 1995. The nervous systems of Cnidarians. In: Breidbach O, Kutsch W, editors. The nervous systems of invertebrates: an evolutionary and comparative approach: with a coda written by T.H. Bullock. Basel: Birkhäuser. p. 7–24.

[bib58] Guo Q, Atkinson SD, Xiao B, Zhai Y, Bartholomew JL, Gu Z. 2022. A myxozoan genome reveals mosaic evolution in a parasitic cnidarian. BMC Biol. 20:51. 35177085 10.1186/s12915-022-01249-8 PMC8855578

[bib59] Hammel I, Lagunoff D, Galli SJ. 2010. Regulation of secretory granule size by the precise generation and fusion of unit granules. J Cellular Molecular Medi. 14:1904–16. 10.1111/j.1582-4934.2010.01071.x PMC2909340 20406331

[bib60] Hausmann K, Holstein T. 1985. Bilateral symmetry in the cnidocil-nematocyst complex of the freshwater medusa*Craspedacusta sowerbii* Lankester (Hydrozoa, Limnomedusae). J Ultrastruct Res. 90:89–104.

[bib61] Havrilak JA, Al-Shaer L, Baban N, Akinci N, Layden MJ. 2021. Characterization of the dynamics and variability of neuronal subtype responses during growth, degrowth, and regeneration of *Nematostella vectensis*. BMC Biol. 19:104. 34001126 10.1186/s12915-021-01038-9 PMC8128482

[bib62] He LS, Qi Y, Allard CA, Valencia-Montoya WA, Krueger SP, Weir K, Seminara A, Bellono NW. 2023. Molecular tuning of sea anemone stinging. elife. 12:RP88900. 37906220 10.7554/eLife.88900 PMC10617991

[bib63] Hehmeyer J, Plessier F, Marlow H. 2024. Adaptive cellular radiations and the genetic mechanisms underlying animal nervous system diversification. Annu Rev Cell Dev Biol. 40:407–25. 39052757 10.1146/annurev-cellbio-111822-124041

[bib64] Hobmayer E, Holstein TW, David CN. 1990. Tentacle morphogenesis in hydra: II. Formation of a complex between a sensory nerve cell and a battery cell. Development. 109:897–904.

[bib65] Hofmann D, Garg N, Grässle S, Vanderheiden S, Bergheim BG, Bräse S, Jung N, Özbek S. 2021. A small molecule screen identifies novel inhibitors of mechanosensory nematocyst discharge in Hydra. Sci Rep. 11:20627. 34663887 10.1038/s41598-021-99974-7 PMC8523708

[bib66] Holstein TW . 2012. A view to kill. BMC Biol. 10:18. 22390773 10.1186/1741-7007-10-18 PMC3293708

[bib67] Holtmann M, Thurm U. 2001. Mono- and oligo-vesicular synapses and their connectivity in a Cnidarian sensory epithelium (*Coryne tubulosa*). J Comparative Neurol. 432:537–49. 10.1002/cne.1118 11268013

[bib68] Horridge GA . 1965. Non-motile sensory cilia and neuromuscular junctions in a ctenophore independent effector organ. Proc R Soc Lond B. 162:333–50.

[bib69] Horridge GA . 1969. Statocysts of medusae and evolution of stereocilia. Tissue Cell. 1:341–53. 18631472 10.1016/s0040-8166(69)80029-7

[bib70] Hundgen M, Biela C. 1982. Fine structure of touch-plates in the scyphomedusan *Aurelia aurita*. J Ultrastruct Res. 80:178–84. 6126597 10.1016/s0022-5320(82)90016-8

[bib71] Hwang JS, Ohyanagi H, Hayakawa S, Osato N, Nishimiya-Fujisawa C, Ikeo K, David CN, Fujisawa T, Gojobori T. 2007. The evolutionary emergence of cell type-specific genes inferred from the gene expression analysis of Hydra. Proc Natl Acad Sci USA. 104:14735–40. 17766437 10.1073/pnas.0703331104 PMC1963347

[bib72] Hwang JS, Takaku Y, Chapman J, Ikeo K, David CN, Gojobori T. 2008. Cilium evolution: identification of a novel protein, nematocilin, in the mechanosensory cilium of hydra nematocytes. Mol Biol Evol. 25:2009–17. 18635678 10.1093/molbev/msn154

[bib73] Hwang JS, Takaku Y, Momose T, Adamczyk P, Ozbek S, Ikeo K, Khalturin K, Hemmrich G, Bosch TCG, Holstein TW et al. 2010. Nematogalectin, a nematocyst protein with GlyXY and galectin domains, demonstrates nematocyte-specific alternative splicing in Hydra. Proc Natl Acad Sci USA. 107:18539–44. 20937891 10.1073/pnas.1003256107 PMC2972925

[bib74] Ispolatov I, Ackermann M, Doebeli M. 2012. Division of labour and the evolution of multicellularity. Proc R Soc B. 279:1768–76. 10.1098/rspb.2011.1999 PMC3297448 22158952

[bib75] Jarman AP, Groves AK. 2013. The role of *Atonal* transcription factors in the development of mechanosensitive cells. Semin Cell Dev Biol. 24:438–47. 23548731 10.1016/j.semcdb.2013.03.010 PMC3778674

[bib76] Jedlicka P, Sui X, Sussel L, Gutierrez-Hartmann A. 2009. Ets transcription factors control epithelial maturation and transit and crypt-villus morphogenesis in the mammalian intestine. Am J Pathol. 174:1280–90. 19264912 10.2353/ajpath.2009.080409 PMC2671360

[bib77] Karabulut A, McClain M, Rubinstein B, Sabin KZ, McKinney SA, Gibson MC. 2022. The architecture and operating mechanism of a cnidarian stinging organelle. Nat Commun. 13:3494. 35715400 10.1038/s41467-022-31090-0 PMC9205923

[bib78] Kass-Simon G, Pierobon P. 2007. Cnidarian chemical neurotransmission, an updated overview. Comp Biochem Physiol A. 146:9–25. 10.1016/j.cbpa.2006.09.008 17101286

[bib79] Kass-Simon G, Scappaticci AA. 2002. The behavioral and developmental physiology of nematocysts. Can J Zool. 80:23.

[bib80] Kelly RB . 1985. Pathways of protein secretion in eukaryotes. Science. 230:25–32. 2994224 10.1126/science.2994224

[bib81] Keramidioti A, Schneid S, Busse C, Cramer von Laue C, Bertulat B, Salvenmoser W, Hess M, Alexandrova O, Glauber KM, Steele RE et al. 2024. A new look at the architecture and dynamics of the Hydra nerve net. eLife. 12:RP87330. 38407174 10.7554/eLife.87330 PMC10942621

[bib82] Kirk DL . 2003. Seeking the ultimate and proximate causes of volvox multicellularity and cellular differentiation. Integr Comp Biol. 43:247–53. 21680429 10.1093/icb/43.2.247

[bib83] Klein O, Roded A, Zur N, Azouz NP, Pasternak O, Hirschberg K, Hammel I, Roche PA, Yatsu A, Fukuda M et al. 2017. Rab5 is critical for SNAP23 regulated granule-granule fusion during compound exocytosis. Sci Rep. 7:15315. 29127297 10.1038/s41598-017-15047-8 PMC5681557

[bib84] Klompen AML, Kayal E, Collins AG, Cartwright P. 2021. Phylogenetic and selection analysis of an expanded family of putatively pore-forming jellyfish toxins (Cnidaria: Medusozoa). Genome Biol Evolut. 13:evab081. 10.1093/gbe/evab081 PMC8214413 33892512

[bib85] Klompen AML, Sanders SM, Cartwright P. 2022. Venom system variation and the division of labor in the colonial hydrozoan Hydractinia symbiolongicarpus. Toxicon: X. 14:100113. 35287376 10.1016/j.toxcx.2022.100113 PMC8917316

[bib86] Klompen AML, Travert MK, Cartwright P. 2023. Localization of multiple jellyfish toxins shows specificity for functionally distinct polyps and nematocyst types in a colonial hydrozoan. Toxins. 15:149. 36828463 10.3390/toxins15020149 PMC9959030

[doi170_899_234525] Koizumi Osamu, Wilson Jeff D., Grimmelikhuijzen Cornelis J. P., Westfall Jane A. 1989. Ultrastructural localization of RFamide‐like peptides in neuronal dense‐cored vesicles in the peduncle of Hydra. Journal of Experimental Zoology, 249:17–22. 10.1002/jez.1402490105 2926357

[bib87] Kraus JEM, Busengdal H, Kraus Y, Hausen H, Rentzsch F. 2024. Doublecortin-like kinase is required for cnidocyte development in *Nematostella vectensis*. Neural Dev. 19:11. 38909268 10.1186/s13064-024-00188-0 PMC11193195

[bib88] Lentz TL, Barrnett RJ. 1965. Fine structure of the nervous system of Hydra. Am Zool. 5:341–56. 14345241 10.1093/icb/5.3.341

[bib89] Leonardi ND, Thuesen Erik V, Haddock SH. 2020. A sticky thicket of glue cells: a comparative morphometric analysis of colloblasts in 20 species of comb jelly (phylum Ctenophora). Cienc. Mar. 46:211–25.

[bib90] Levy S, Elek A, Grau-Bové X, Menéndez-Bravo S, Iglesias M, Tanay A, Mass T, Sebé-Pedrós A. 2021. A stony coral cell atlas illuminates the molecular and cellular basis of coral symbiosis, calcification, and immunity. Cell. 184:2973–2987.e18. 33945788 10.1016/j.cell.2021.04.005 PMC8162421

[bib91] Lovas JR, Yuste R. 2021. Ensemble synchronization in the reassembly of *Hydra*’s nervous system. Curr Biol. 31:3784–3796.e3. 34297913 10.1016/j.cub.2021.06.047

[bib92] Maday S, Twelvetrees AE, Moughamian AJ, Holzbaur ELF. 2014. Axonal transport: cargo-specific mechanisms of motility and regulation. Neuron. 84:292–309. 25374356 10.1016/j.neuron.2014.10.019 PMC4269290

[bib93] Mahoney JL, Graugnard EM, Mire P, Watson GM. 2011. Evidence for involvement of TRPA1 in the detection of vibrations by hair bundle mechanoreceptors in sea anemones. J Comp Physiol A. 197:729–42. 10.1007/s00359-011-0636-7 21394510

[bib94] Marino A, Morabito R, La Spada G, Adragna NC, Lauf PK. 2011. Evidence for aquaporin-mediated water transport in nematocytes of the jellyfish Pelagia noctiluca. Cell Physiol Biochem. 28:1211–8. 22179009 10.1159/000335853

[bib96] Mariscal RN, Bigger CH, McLean RB. 1976. The form and function of cnidarian spirocysts 1. Ultrastructure of the capsule exterior and relationship to the tentacle sensory surface. Cell Tissue Res. 168:465–74. 6149 10.1007/BF00215997

[bib95] Mariscal RN . 1974. Nematocysts. In: Muscatine L, editor. Coelenterate biology: reviews and new perspectives. New York: Academic Press. p. 129–78.

[bib97] McAfee JS, Benson C, Spangenberg D, Lattanzio F, Strasnick B. 2015. Jellyfish model for ototoxicity. Otol Neurotol. 36:329. 24786541 10.1097/MAO.0000000000000402

[bib98] McDonald JMC, Reed RD. 2024. Beyond modular enhancers: new questions in *cis-*regulatory evolution. Trends Ecol Evol. 39:1035–46. 39266441 10.1016/j.tree.2024.07.005

[bib99] McShane A, Bath J, Jaramillo AM, Ridley C, Walsh AA, Evans CM, Thornton DJ, Ribbeck K. 2021. Mucus. Curr Biol. 31:R938–45. 34375594 10.1016/j.cub.2021.06.093 PMC8759706

[bib100] Menard SS, Watson GM. 2017. Evidence for two populations of hair bundles in the sea anemone, *Nematostella vectensis*. Comp Biochem Physiol A. 208:14–23. 10.1016/j.cbpa.2017.03.006 28315771

[bib101] Mire-Thibodeaux P, Watson GM. 1994. Morphodynamic hair bundles arising from sensory cell/supporting cell complexes frequency-tune nematocyst discharge in sea anemones. J Exp Zool. 268:282–92. 8195744 10.1002/jez.1402680404

[bib102] Mironov AA, Arvan P. 2008. Origins of the regulated secretory pathway. In: The Golgi Apparatus. A Mironov, ​​​​M Pavelkaeditors. 485–519.

[bib103] Moran Y, Praher D, Schlesinger A, Ayalon A, Tal Y, Technau U. 2013. Analysis of soluble protein contents from the nematocysts of a model sea anemone sheds light on venom evolution. Mar Biotechnol. 15:329–39. 10.1007/s10126-012-9491-y PMC3627010 23151943

[bib104] Morris SA . 2019. The evolving concept of cell identity in the single cell era. Development. 146:dev169748. 31249002 10.1242/dev.169748

[bib105] Musser JM, Schippers KJ, Nickel M, Mizzon G, Kohn AB, Pape C, Ronchi P, Papadopoulos N, Tarashansky AJ, Hammel JU et al. 2021. Profiling cellular diversity in sponges informs animal cell type and nervous system evolution. Science. 374:717–23. 34735222 10.1126/science.abj2949 PMC9233960

[bib106] Najle SR, Grau-Bové X, Elek A, Navarrete C, Cianferoni D, Chiva C, Cañas-Armenteros D, Mallabiabarrena A, Kamm K, Sabidó E et al. 2023. Stepwise emergence of the neuronal gene expression program in early animal evolution. Cell. 186:4676–4693.e29. 37729907 10.1016/j.cell.2023.08.027 PMC10580291

[bib107] Norekian TP, Meech RW. 2020. Structure and function of the nervous system in nectophores of the siphonophore Nanomia bijuga. J Exp Biol. 223:jeb233494. 33168595 10.1242/jeb.233494

[bib108] Oliver D, Brinkmann M, Sieger T, Thurm U. 2008. Hydrozoan nematocytes send and receive synaptic signals induced by mechano-chemical stimuli. J Exp Biol. 211:2876–88. 18723547 10.1242/jeb.018515

[bib110] Özbek S, Balasubramanian PG, Holstein TW. 2009. Cnidocyst structure and the biomechanics of discharge. Toxicon. 54:1038–45. 19286000 10.1016/j.toxicon.2009.03.006

[bib109] Özbek S . 2011. The cnidarian nematocyst: a miniature extracellular matrix within a secretory vesicle. Protoplasma. 248:635–40. 20957500 10.1007/s00709-010-0219-4

[bib111] Ozment E, Tamvacakis AN, Zhou J, Rosiles-Loeza PY, Escobar-Hernandez EE, Fernandez-Valverde SL, Nakanishi N. 2021. Cnidarian hair cell development illuminates an ancient role for the class IV POU transcription factor in defining mechanoreceptor identity. eLife. 10:e74336. 34939935 10.7554/eLife.74336 PMC8846589

[bib112] Pantin CFA . 1942. The excitation of nematocysts. Nature. 149; 109.

[bib113] Peteya DJ . 1975. The ciliary-cone sensory cell of anemones and cerianthids. Tissue Cell. 7:243–52. 238306 10.1016/0040-8166(75)90003-8

[bib114] Picciani N, Kerlin JR, Jindrich K, Hensley NM, Gold DA, Oakley TH. 2021. Light modulated cnidocyte discharge predates the origins of eyes in Cnidaria. Ecol Evol. 11:3933–40. 33976785 10.1002/ece3.7280 PMC8093662

[bib115] Pickett JA, Edwardson JM. 2006. Compound exocytosis: mechanisms and functional significance. Traffic. 7:109–16. 16420520 10.1111/j.1600-0854.2005.00372.x

[bib116] Piriatinskiy G, Atkinson SD, Park S, Morgenstern D, Brekhman V, Yossifon G, Bartholomew JL, Lotan T. 2017. Functional and proteomic analysis of *Ceratonova shasta* (Cnidaria: Myxozoa) polar capsules reveals adaptations to parasitism. Sci Rep. 7:9010. 28827642 10.1038/s41598-017-09955-y PMC5566210

[bib117] Plachetzki DC, Fong CR, Oakley TH. 2012. Cnidocyte discharge is regulated by light and opsin-mediated phototransduction. BMC Biol. 10:17. 22390726 10.1186/1741-7007-10-17 PMC3329406

[bib118] Pomaville MB, Sattler SM, Abitua PB. 2024. A new dawn for the study of cell type evolution. Development. 151:dev200884. 38722217 10.1242/dev.200884 PMC11128286

[bib119] Rachamim T, Morgenstern D, Aharonovich D, Brekhman V, Lotan T, Sher D. 2015. The dynamically evolving nematocyst content of an anthozoan, a scyphozoan, and a hydrozoan. Mol Biol Evol. 32:740–53. 25518955 10.1093/molbev/msu335

[bib120] Rentzsch F, Juliano C, Galliot B. 2019. Modern genomic tools reveal the structural and cellular diversity of cnidarian nervous systems. Curr Opin Neurobiol. 56:87–96. 30654234 10.1016/j.conb.2018.12.004

[bib121] Rentzsch F, Layden M, Manuel M. 2017. The cellular and molecular basis of cnidarian neurogenesis: cnidarian neurogenesis: cellular and molecular basis. WIREs Dev Biol. 6:e257. 10.1002/wdev.257 PMC6680159 27882698

[bib122] Richards GS, Rentzsch F. 2014. Transgenic analysis of a *SoxB* gene reveals neural progenitor cells in the cnidarian *Nematostella vectensis*. Development. 141:4681–9. 25395455 10.1242/dev.112029

[bib123] Richards GS, Rentzsch F. 2015. Regulation of *Nematostella* neural progenitors by SoxB, Notch and bHLH genes. Development. 142:3332–42. 26443634 10.1242/dev.123745 PMC4631755

[bib124] Ryan JF, Chiodin M. 2015. Where is my mind? How sponges and placozoans may have lost neural cell types. Phil Trans R Soc B. 370:20150059. 26554046 10.1098/rstb.2015.0059 PMC4650130

[bib125] Salamanca-Díaz DA, Horkan HR, García-Castro H, Emili E, Salinas-Saavedra M, Pérez-Posada A, Rossi ME, Álvarez-Presas M, Mac Gabhann R, Hillenbrand P et al. 2025. The Hydractinia cell atlas reveals cellular and molecular principles of cnidarian coloniality. Nat Commun. 16:2121. 40032860 10.1038/s41467-025-57168-z PMC11876637

[bib126] Salamanca-Díaz DA, Ritschard EA, Schmidbaur H, Wanninger A. 2022. Comparative single-cell transcriptomics reveals novel genes involved in bivalve embryonic shell formation and questions ontogenetic homology of molluscan shell types. Front Cell Dev Biol. 10:883755. 35813198 10.3389/fcell.2022.883755 PMC9261976

[bib127] Schnitzler CE, Chang ES, Waletich J, Quiroga-Artigas G, Wong WY, Nguyen A-D, Barreira SN, Doonan LB, Gonzalez P, Koren S et al. 2024. The genome of the colonial hydroid Hydractinia reveals that their stem cells use a toolkit of evolutionarily shared genes with all animals. Genome Res. 34:498–513. 38508693 10.1101/gr.278382.123 PMC11067881

[bib128] Sebé-Pedrós A, Saudemont B, Chomsky E, Plessier F, Mailhé M-P, Renno J, Loe-Mie Y, Lifshitz A, Mukamel Z, Schmutz S et al. 2018. Cnidarian cell type diversity and regulation revealed by whole-organism single-cell RNA-Seq. Cell. 173:1520–1534.e20. 29856957 10.1016/j.cell.2018.05.019

[bib129] Senatore A, Raiss H, Le P. 2016. Physiology and evolution of voltage-gated calcium channels in early diverging animal phyla: Cnidaria, Placozoa, Porifera and Ctenophora. Front Physiol. 7: 481. 27867359 10.3389/fphys.2016.00481 PMC5095125

[bib130] Serb JM, Oakley TH. 2005. Hierarchical phylogenetics as a quantitative analytical framework for evolutionary developmental biology. Bioessays. 27:1158–66. 16237676 10.1002/bies.20291

[bib131] Shpirer E, Diamant A, Cartwright P, Huchon D. 2018. A genome wide survey reveals multiple nematocyst-specific genes in Myxozoa. BMC Evol Biol. 18:138. 30208843 10.1186/s12862-018-1253-7 PMC6134521

[bib132] Siebert S, Farrell JA, Cazet JF, Abeykoon Y, Primack AS, Schnitzler CE, Juliano CE. 2019. Stem cell differentiation trajectories in *Hydra* resolved at single-cell resolution. Science. 365:eaav9314. 31346039 10.1126/science.aav9314 PMC7104783

[bib133] Sierra NC, Gold DA. 2024. The evolution of cnidarian stinging cells supports a Precambrian radiation of animal predators. Evol Dev. 26:e12469. 38236185 10.1111/ede.12469

[bib134] Singla CL . 1975. Statocysts of hydromedusae. Cell Tissue Res. 158: 391–407. 238743 10.1007/BF00223835

[bib135] Smith S, Oshida J, Bode H. 1974. Inhibition of nematocyst discharge in Hydra fed to repletion. Biol Bull. 147:186–202.

[bib136] Sprecher SG . 2022. Neural cell type diversity in Cnidaria. Front Neurosci. 24: 909400. 10.3389/fnins.2022.909400 PMC9170993 35685775

[bib137] Steger J, Cole AG, Denner A, Lebedeva T, Genikhovich G, Ries A, Reischl R, Taudes E, Lassnig M, Technau U. 2022. Single-cell transcriptomics identifies conserved regulators of neuroglandular lineages. Cell Rep. 40:111370. 36130520 10.1016/j.celrep.2022.111370

[bib138] Stidwill RP, Honegger TG. 1989. A single layer of microtubules is part of a complex cytoskeleton in mature nematocytes of hydra. Tissue Cell. 21:179–88. 18620258 10.1016/0040-8166(89)90062-1

[bib139] Sunagar K, Columbus-Shenkar YY, Fridrich A, Gutkovich N, Aharoni R, Moran Y. 2018. Cell type-specific expression profiling unravels the development and evolution of stinging cells in sea anemone. BMC Biol. 16:108. 30261880 10.1186/s12915-018-0578-4 PMC6161364

[bib140] Surm jM, landau M, columbus-shenkar yY, moran Y. 2024. sea anemone membrane attack complex/perforin superfamily demonstrates an evolutionary transitional state between venomous and developmental functions. mol biol evol. 41:msae082. 38676945 10.1093/molbev/msae082 PMC11090067

[bib141] tang P-C, Watson GM. 2014. Cadherin-23 May be dynamic in hair bundles of the model sea anemone *Nematostella vectensis*. PLoS One. 9:e86084. 24465885 10.1371/journal.pone.0086084 PMC3899209

[bib142] Tang P-C, Watson GM. 2015. Proteomic identification of hair cell repair proteins in the model sea anemone *Nematostella vectensis*. Hear Res. 327:245–56. 26183436 10.1016/j.heares.2015.07.005

[bib144] Tardent P, Schmid V. 1972. Ultrastructure of mechanoreceptors of the polyp *Coryne pintneri* (Hydrozoa, Athecata). Exp Cell Res. 72:265–75. 4402012 10.1016/0014-4827(72)90589-7

[bib143] Tardent P . 1995. The cnidarian cnidocyte, a hightech cellular weaponry. Bioessays. 17:351–62.

[bib145] Thomas J, Morlé L, Soulavie F, Laurençon A, Sagnol S, Durand B. 2010. Transcriptional control of genes involved in ciliogenesis: a first step in making cilia. Biol Cell. 102:499–513. 20690903 10.1042/BC20100035

[bib146] Thorington GU, McAULEY V, Hessinger DA 2010. Effects of satiation and starvation on nematocyst discharge, prey killing, and ingestion in two species of sea anemone. Biol Bull. 219:122–31. 20972257 10.1086/BBLv219n2p122

[bib147] Thorn P, Gaisano H. 2012. Molecular control of compound Exocytosis. Commun Integrative Biol. 5:61–3. 10.4161/cib.18058 PMC3291316 22482012

[bib148] Thurm U, Brinkmann M, Golz R, Holtmann M, Oliver D, Sieger T. 2004. Mechanoreception and synaptic transmission of hydrozoan nematocytes. Hydrobiologia. 530:97–105.

[bib149] Tournière O, Dolan D, Richards GS, Sunagar K, Columbus-Shenkar YY, Moran Y, Rentzsch F. 2020. NvPOU4/Brain3 functions as a terminal selector gene in the nervous system of the cnidarian *Nematostella vectensis*. Cell Rep. 30:4473–4489.e5. 32234481 10.1016/j.celrep.2020.03.031

[bib150] Van Oss SB, Carvunis A-R. 2019. De novo gene birth. PLoS Genet. 15:e1008160. 31120894 10.1371/journal.pgen.1008160 PMC6542195

[bib151] Watanabe H, Fujisawa T, Holstein TW. 2009. Cnidarians and the evolutionary origin of the nervous system: cnidarian nervous system. Dev Growth Differ. 51:167–83. 19379274 10.1111/j.1440-169X.2009.01103.x

[bib152] Watson GM, Graugnard EM, Mire P. 2007. The involvement of Arl-5b in the repair of hair cells in sea anemones. JARO. 8:183–93. 17332968 10.1007/s10162-007-0078-7 PMC2538354

[bib153] Watson GM, Hessinger DA. 1987. Receptor-mediated endocytosis of a chemoreceptor involved in triggering the discharge of cnidae in a sea anemone tentacle. Tissue Cell. 19:747–55. 18620220 10.1016/0040-8166(87)90016-4

[bib154] Watson GM, Hessinger DA. 1989. Cnidocytes and adjacent supporting cells form receptor-effector complexes in anemone tentacles. Tissue Cell. 21:17–24. 18620251 10.1016/0040-8166(89)90017-7

[bib155] Watson GM, Mire P, Hudson RR. 1997. Hair bundles of sea anemones as a model system for vertebrate hair bundles. Hear Res. 107:53–66. 9165347 10.1016/s0378-5955(97)00022-1

[bib156] Watson GM, Mire P, Kinler KM. 2009. Mechanosensitivity in the model sea anemone *Nematostella vectensis*. Mar Biol. 156:2129–37.

[bib157] Watson GM, Mire-Thibodeaux P. 1994. The cell biology of nematocysts. In: Jeon KW, Jarvik J, editors. International review of cytology. Cambridge: Academic Press. p. 275–300. 10.1016/s0074-7696(08)62256-1 7860218

[bib158] Watson GM, Roberts J. 1994. Localization of proline receptors involved in regulating nematocyst discharge. J Exp Zool. 270:527–37.

[bib159] Watson GM, Roberts J. 1995. Chemoreceptor-mediated polymerization and depolymerization of actin in hair bundles of sea anemones. Cell Motil Cytoskeleton. 30:208–20. 7758137 10.1002/cm.970300305

[bib160] Weir K, Dupre C, van Giesen L, Lee AS-Y, Bellono NW. 2020. A molecular filter for the cnidarian stinging response. eLife. 9:e57578. 32452384 10.7554/eLife.57578 PMC7250568

[doi171_213_230025] Westfall J. A., Grimmelikhuijzen CJP. 1993. Antho-RFamide Immunoreactivity in Neuronal Synaptic and Nonsynaptic Vesicles of Sea Anemones. The Biological Bulletin, 185:109–114. 10.2307/1542134 29300595

[bib164] Westfall JA, Elliott CF, Carlin RW. 2002. Ultrastructural evidence for two-cell and three-cell neural pathways in the tentacle epidermis of the sea anemoneAiptasia pallida. J Morphol. 251:83–92. 11746469 10.1002/jmor.1075

[bib165] Westfall JA, Sayyar KL, Elliott CF. 1998. Cellular origins of kinocilia, stereocilia, and microvilli on tentacles of sea anemones of the genus Calliactis (Cnidaria: Anthozoa). Invertebrate Biol. 117:186.

[bib161] Westfall JA . 1970. The nematocyte complex in a hydromedusan, *Gonionemus vertens*. Z. Zellforsch. 110:457–70. 4396767 10.1007/BF00330098

[bib162] Westfall JA . 1973. Ultrastructural evidence for a granule-containing sensory-motor-interneuron in *Hydra littoralis*. J Ultrastruct Res. 42:268–82. 4702922 10.1016/s0022-5320(73)90055-5

[bib163] Westfall JA . 1996. Ultrastructure of synapses in the first-evolved nervous systems. J Neurocytol. 25:735–46. 9023721 10.1007/BF02284838

[bib166] Weston AJ, Chung R, Dunlap WC, Morandini AC, Marques AC, Moura-da-Silva AM, Ward M, Padilla G, da Silva LF, Andreakis N et al. 2013. Proteomic characterisation of toxins isolated from nematocysts of the South Atlantic jellyfish Olindias sambaquiensis. Toxicon. 71:11–7. 23688393 10.1016/j.toxicon.2013.05.002

[bib167] Yu S-M, Westfall JA, Dunne JF. 1985. Light and electron microscopic localization of a monoclonal antibody in neurons in situ in the head region of Hydra. J Morphol. 184:183–93. 3989866 10.1002/jmor.1051840208

[bib168] Zeng H . 2022. What is a cell type and how to define it?. Cell. 185:2739–55. 35868277 10.1016/j.cell.2022.06.031 PMC9342916

[bib169] Zenkert C, Takahashi T, Diesner M-O, Özbek S. 2011. Morphological and molecular analysis of the *Nematostella vectensis* cnidom. PLoS One. 6:e22725. 21829492 10.1371/journal.pone.0022725 PMC3145756

